# Expression of a *Deschampsia antarctica* Desv. Polypeptide with Lipase Activity in a *Pichia pastoris* Vector

**DOI:** 10.3390/ijms15022359

**Published:** 2014-02-07

**Authors:** Claudia Rabert, Ana Gutiérrez-Moraga, Alejandro Navarrete-Gallegos, Darío Navarrete-Campos, León A. Bravo, Manuel Gidekel

**Affiliations:** 1 Laboratorio de Fisiología y Biología Molecular Vegetal, Instituto de Agroindustria, Facultad de Ciencias Agropecuarias y Forestales, Universidad de La Frontera, Casilla 54D, Temuco 4811230, Chile; E-Mails: claudia.rabert@ufrontera.cl (C.R.); hilda.gutierrez@ufrontera.cl (A.G.-M.); leon.bravo@ufrontera.cl (L.A.B.); 2 Center of Plant, Soil Interaction and Natural Resources Biotechnology, Scientific and Technological Bioresource Nucleus, Universidad de La Frontera, Casilla 54D, Temuco 4811230, Chile; 3 Laboratorio de Fisiología Vegetal, Departamento de Botánica, Universidad de Concepción, Casilla 160-C, Correo 3, Concepción 3801061, Chile; E-Mails: anavarre@gmail.com (A.N.-G.); darnacos@gmail.com (D.N.-C.); 4 Vicerrectoría de Investigación y Postgrado, Universidad de La Frontera, Casilla 54D, Temuco 4811230, Chile

**Keywords:** *Deschampsia antarctica*, lipase, *Pichia pastoris*, detergent tolerance

## Abstract

The current study isolated and characterized the Lip3F9 polypeptide sequence of *Deschampsia antarctica* Desv. (GeneBank Accession Number JX846628), which was found to be comprised of 291 base pairs and was, moreover, expressed in *Pichia pastoris* X-33 cells. The enzyme was secreted after 24 h of *P. pastoris* culture incubation and through induction with methanol. The expressed protein showed maximum lipase activity (35 U/L) with an optimal temperature of 37 °C. The lipase-expressed enzyme lost 50% of its specific activity at 25 °C, a behavior characteristic of a psychrotolerant enzyme. Recombinant enzyme activity was measured in the presence of ionic and non-ionic detergents, and a decrease in enzyme activity was detected for all concentrations of ionic and non-ionic detergents assessed.

## 1. Introduction

The Antarctic continent is considered one of the harshest ecosystems in the world [1]. Isolation and environmental history have led to a unique biota, both on land and in the sea [2]. The Antarctic hair grass *Deschampsia antarctica* Desv. and the cushion-forming pearlwort *Colobanthus quitensis* are the only two native, vascular plants that can tolerate these harsh environmental conditions and survive south of 56°S [3].

Many researchers are interested in *D. antarctica* due to its ability to live in these extreme conditions, and there are an increasing number of physiological and biochemical studies being undertaken to better understand the mechanisms by which this plant is capable of colonizing the Antarctic environment [4]. *D. antarctica* is a perennial grass distributed continuously from 68°43′ to 67°32′W along the Antarctic coastline, and the greatest abundances are found at Lynch Island, South Orkney Island, South Shetland Island, and South Sandwich Island [5].

Lipases (triacylglycerol hydrolases, EC 3.1.1.3.) [6] are classes of enzymes which catalyze the hydrolysis of long chain triglycerides, and these constitute the most important group of biocatalysts for biotechnological applications [7]. The GDSL motif enzyme is a relatively newly discovered lipase, share five blocks of highly conserved homology, which are important for their classification and the active-site Ser is located close to the *N*-terminus [8], are widely exist in both microbe and plant species that participate in the hydrolysis and synthesis of lipids or esters [9]. However, information about the extraction or expression of enzymes from *D. antarctica* is nonexistent, and only one gene has been isolated and cloned in the pGapαA expression vector of *P. pastoris*. This protein showed increased activity when the plant was exposed to a temperature of 4 °C and UV radiation [10,11].

The identification and characterization of genes coding for lipase-like proteins in plant species tolerant to low temperatures, such as *D. antarctica*, can provide novel enzymes for industrial applications. To this end, this study is the first report to isolate and characterize a polypeptide with lipase activity from *D. antarctica* through expression in the pPICZαB *Pichia pastoris* vector.

## 2. Results and Discussion

### 2.1. Isolation and Analysis of the Lip3F9 Polypeptide

**Figure 1.**
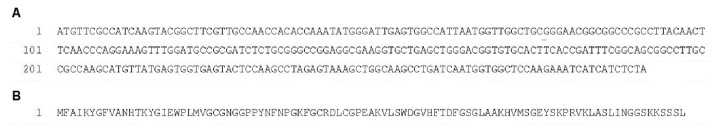
Sequences of the Lip3F9 polypeptide of *D. antarctica*. (**A**) The nucleotide sequence and (**B**) The deduced amino acid sequence is shown as a one letter code (GeneBank Accession Number JX846628).

The sequence labeled Lip3F9, coding for a lipase-like enzyme (triglyceride lipases EC 3.1.1.3), was identified through an analysis of a cDNA expression library from leaf samples of *D. antarctica*. The sequence was comprised of 291 nucleotides encoding for a protein composed of 97 amino acids (Figure 1). This gene sequence was compared with other available sequences in the public NCBI Genebank through the BLASTX program. The cDNA of Lip3F9 presented a full-length match with NP_001054716.2, a sequence identified as a putative GDSL lipase/acylhydrolase of *Orysa sativa* (japonica cultivar-group), where the percent of sequence identity was 56/101 (56%) and the percent of positive substitutions was 70/101 (70%).

A search against the Conserved Domains Database (CCD) revealed that the Lip3F9 amino acid sequence possessed conserved domains in the SGNH hydrolase superfamily (cl01053) and specifically in SGNH plant lipase-like proteins (cd01837), all of which contain a conserved GDSL domain (pfam00657).

Analysis of the Lip3F9 sequence revealed that the polypeptide corresponded to the extreme carboxyl terminal (*C*-terminal) of the protein sequence, and the presence of Asp58 and His61, two out of three amino acids for the catalytic triad and active sites were detected.

### 2.2. Expression of the Lip3F9 Polypeptide in *Pichia pastoris*

The Lip3F9 polypeptide sequence was cloned into the pPICZαB pichia expression vector. The resulting plasmid construct, pPICZαB–Lip3F9, was transformed into *E. coli*, and transformants were selected on a low salt LB medium with Zeocin. The recombinant plasmid was sequenced with the 5-AOX1 sequencing primer (5′ GACTGGTTCCAATTGACAAGC) to confirm the open reading frame during later sequencing. The correct arrangement of signaling, gene, and *C*-terminal polyhistidine tag elements in the constructed sequence were checked, and enough plasmid DNA was extracted for transformation into the methylotrophic yeast *Pichia pastoris*, wild type host strain X-33. The purified pPICZαB–Lip3F9 plasmid was digested with *Sac*I (Fermentas, Burlington, ON, Canada), and after verifying complete digestion by agarose gel electrophoresis, transformation was performed. Eleven colonies of Zeocin resistant, recombinant yeast were obtained. Gene insertion was checked in all clones through PCR analysis with the primers 5-AOX1 and reverse Lip-Da, and transformants were checked for methanol utilization by plating onto minimal methanol (MM) and a minimal dextrose (MD) plates. All transformants presented a Mut^+^ phenotype (not shown).

Through software analysis with Vector NTI Advance 11 (Invitrogen, Carlsbad, CA, USA), *Pichia pastoris* was estimated to express a molecular protein weight of 10.3 kDa and an isoelectric point of 8.99. These estimates were different from other parameters reported in lipases, most of which present molecular masses between 27 and 60 kDa and isoelectric points between 3.5 and 7.3 [12]. These differences could be attributed to working with a polypeptide sequence.

Several lipases have been obtained by cloning into expression vectors from *Pichia pastoris*, among which the expression of *Candida antarctica* lipase B [13], the expression and characterization of *Candida rugosa* lipase 4 [14], and the expression, purification, and characterization of *Yarrowia lipolytic* lipase 2 [15] have been achieved. Although there are various studies which have used vector cloning methods for expression analysis, it would be false to assume that poor investigation methods are to fault for *P. pastoris* derived expression analyses of lipases from microorganisms and vascular plants. The majority of plant lipases have been obtained from seeds through laborious methodologies based on traditional purification strategies [16], and the *P. pastoris* extraction system is used when no information is available for the nucleotide sequences that allow lipase expression. In the present study, information on Lip3F9 was available through the cDNA expression library.

The activity of the heterologous protein of the Lip3F9 polypeptide was determined in 11 recombinant yeast clones. The 11 clones secreted the recombinant lipase into the medium, and maximum lipase activity was obtained with clone no. 3 and recorded as 8.63 U/L. Control transformants were induced with methanol and did not show lipase activity in the medium (data not shown), demonstrating that the expressed polypeptide is a functional domain with lipase activity. Lipase activity of the KLB1 lipase in the *Pseudomonas* sp. was reported as 7.11 U/mL [17]. While this activity is greater than the activity presented in the clone for Lip3F9, this difference can be attributed to the differing methodologies applied for activity measurement in each study.

A minimum catalytic site with enzymatic activity for water-soluble substrates has been reported in the *C*-terminal of human and porcine pancreatic lipases and other lipases from microbes [12]. A peptide chain fragment of porcine pancreatic lipase that has enzymatic activity in the *p*-nitrophenyl acetate of 114 amino acids has been reported to act as a functional domain of lipase [18]. These results coincide with those obtained in this investigation.

### 2.3. Determination of Range and Optimum Temperature

**Figure 2.**
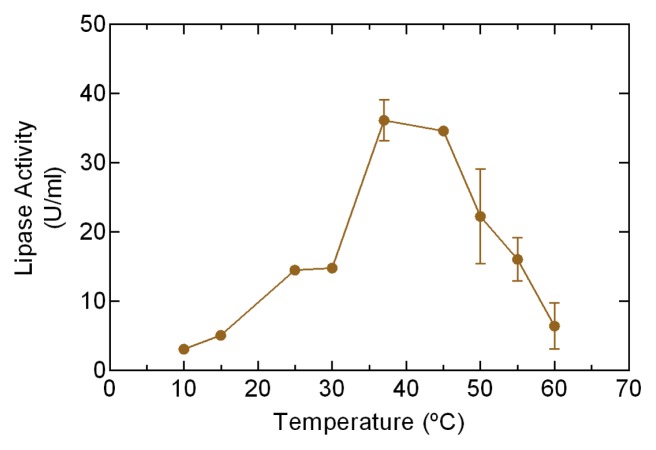
Evaluation of enzymatic activity in a crude extract. Lipolytic activity was measured spectrophotometrically at 96 h post induction of genes in the *P. pastoris* culture with methanol. The enzymatic activity was assayed with temperatures ranging from 10 to 60 °C. The errors bars represent SD values for 3 replicates.

In order to determine the optimum temperature for lipase activity, recombinant protein samples were obtained from the cultures after 96 h of induction (Figure 2). A maximum lipase activity of 35 U/L was observed at 37 °C, whereas 50% of the activity was lost at 25 °C. This result is concordant with previous reports in other plant lipases, such as the lipase isolated from the seed of *Cocos nucifera* linn which had maximum activity at 35 °C [19]. The most important characteristic observed in Lip3F9 clones was the capacity to maintain 50% activity at 25 °C. This behavior is characteristic of a psychrotolerant enzyme, which is consistent with the physiological parameters measured and reported in *D. antarctica.*

### 2.4. Determination of Tolerance Detergent

**Figure 3.**
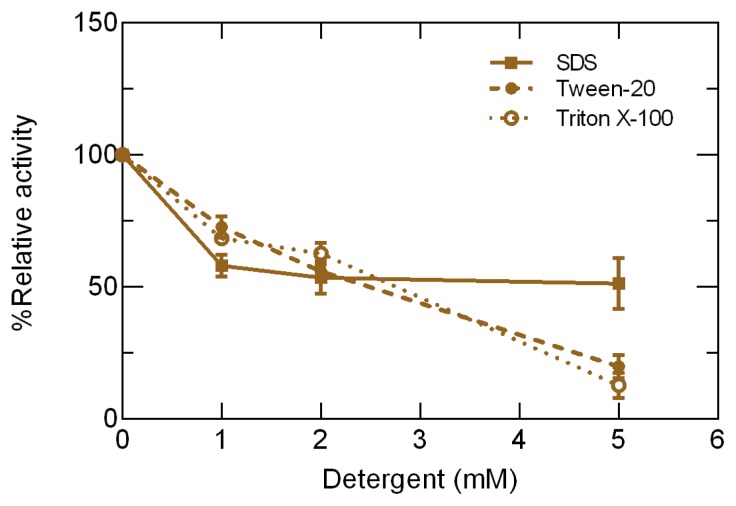
Lipase activity inhibition in the presence of detergents. Lipase activity was measured in the presence of increasing concentrations of the ionic detergent SDS and non-ionic detergents Tween-20 and Triton X-100. The errors bars represent SD values for three replicates.

This evaluation was used to assess enzyme sensitivity to the presence of detergents commonly used in industrial applications and to examine the potential use of the Lip3F9 lipase as an additive for cleaning formulas. Figure 3 demonstrates that all detergents with concentrations of 1 mM caused a 33% decline in initial activity. In the case of the cationic detergent sodium dodecyl sulfate (SDS), activity loss was stabilized with high concentrations (2–5 mM), whereas non-ionic detergents Triton X-100 and Tween-20 produced a constant inhibition of lipase activity. The detergent with the greatest inhibition at high concentrations was Triton X-100, while at lower concentrations it was SDS. Comparatively, the inhibition of enzyme activity in the presence of a detergent was lower for the ionic detergent and similar for non-ionic detergents. These results are consistent with the proposal that the influence of these compounds on lipase activity is dose dependent [20]. A characterization of the inhibition caused by different surfactants was carried out in lipase from rice bran, and the results differed from those obtained with Lip3F9. For rice bran lipase, a 2 mM concentration of SDS caused the greatest inhibitory at 50%, while Triton X-100 had an inhibitory effect at a low concentration of 0.5 mM and restored activity when in a higher concentration of 4 mM [21]. Together with previous studies, the results shown in the present study confirm that the effects of detergents are not homogenous for all enzymes nor are enzymes affected in the same way by all surfactants [22].

## 3. Experimental Section

### 3.1. Screening of the cDNA Library for *D. antarctica*

A cDNA library for *D. antarctica*, cloned into the vector pEXP AD-502 (Invitrogen, Carlsbad, CA, USA), is maintained by the Laboratory of Applied Molecular Biology at the University of La Frontera, Chile. Screening was done through amplification of the inserts using the primers FW2654 (5′ GTACAAAAAAGCAGGCTTGTCG 3′) and REV2655 (5′ GTACAAGAAAGCTGGGTACG 3′). A subsequent search for identity with the National Center for Biotechnology Information (NCBI) database was performed.

### 3.2. Sequence Analysis

Sequence alignment, translation of the open reading frame, and calculation of the predicted molecular mass for proteins were performed using the Vector NTI Advance 11.0 program (Invitrogen, Carlsbad, CA, USA). Searched for homology were carried out using the BLAST program (http://www.ncbi.nlm.nih.gov/blast/Blast.cgi) accessed through the NCBI web platform. DNA sequencing was performed on an ABI 3730XL sequencer (Applied Biosystems, Foster City, CA, USA) by Macrogen (Macrogen Inc., Seoul, Korea).

### 3.3. Vector Construction and Transformation

The polypeptide Lip3F9 was isolated by polymerase chain reaction (PCR) amplification using the primers fwLipDa (5′ CATGTTCGCCATCAAGTACG) and revLipDa (5′ TCTAGAGATGATGA TTTCTTGGAGC). For the reverse LipDa primer, an *Xba*I restriction site was introduced. All PCR amplifications were performed using Taq DNA Polymerase (recombinant), LC (Fermentas, Burlington, ON, Canada). PCR fragments were purified from agarose gels using the UltraClean^®^ 15 DNA Purification Kit (MO BIO Laboratories, Inc., Carlsbad, CA, USA). DNA was purified and manipulated as essentially described by Sambrook *et al.* [23]. The PCR product was cloned into pGEM-T Easy (Promega, Madison, WI, USA), excised with the enzymes *EcoR*I and *Xba*I, and then ligated to the pPICZαB vector digested with the same restriction enzymes. This resulted in the expression vector pPICZαB-Lip3F9. The resulting plasmid constructs were transformed into *E. coli*, and transformants were selected on a low salt LB medium with Zeocin. The recombinant plasmid was sequenced with the 5-AOX1 sequencing primer (5′ GACTGGTTCCAATTGACAAGC). Sequence analysis was performed using the BLAST algorithm.

### 3.4. Lipase Expression in *Pichia pastoris*

Electrocompetent cells of *P. pastoris* X-33 were prepared according to the supplier’s instructions (Invitrogen, Carlsbad, CA, USA). Ten micrograms of the recombinant linearized plasmid were mixed with 80 μL of electrocompetent cells and electroporated through a pulse discharge (1500 V, 25 F, Bio-Rad Gene Pulser, Hercules, CA, USA) for 5 ms. After pulsing, 1 mL of ice-cold sorbitol (1 M) was immediately added to the cuvette. Then, 200 μL aliquots were spread on YPDS plates (1% yeast extract, 2% peptone, 2% dextrose, 1 M sorbitol, 2% agar, 100 μg/mL Zeocin), and the plates were incubated at 30 °C to screen for Zeocin resistant transformants. Zeocin resistant clones were grown on a buffered glycerol-complex (BMGY) medium at 30 °C overnight until *OD*_600_ = 2–6. Finally, clones were transferred to a buffered methanol-complex (BMMY) medium.

### 3.5. Induction of pPICZαB-Lip3F9 Expression

The *P. pastoris* cultures were grown in the BMMY medium for 96 h and centrifuged at 2500 rpm. The resulting pellet was resuspended in the inductor medium containing 2% peptone, 1% yeast extract, and 1.34% yeast nitrogen base (YNB), 0.5% methanol, 100 mM of buffer pH 6.0, and 4 × 10^−5^% biotin to induce the expression of the inserted gene. Every 24 h the medium was supplemented with methanol to maintain concentration. For evaluating all of the clones obtained after 120 h of growth, an induction medium was used. For evaluating the optimum temperature for enzymatic activity at 24, 48, 72 and 96 h, supernatant samples were collected through centrifugation at 2500 rpm post-induction. Aliquots of supernatant were taken, centrifuged for 10 min at 13,000× *g*, and filtered through membranes with a pore diameter of 0.22 μm. With this procedure, a cell-free extract was obtained that was absolutely and totally clarified.

### 3.6. Measurement of Lipase Activity

Hydrolytic activity of the heterologous enzyme lipase was measured using the Lipase Kit DC FS (Diagnostic Systems International, Holzheim, Germany) according to manufacturer’s instructions. The kit is based on the use of a synthetic substrate for lipase (1,2-*O*-dilauryl rac-glycero-3-glutaric acid-(6-methylresorufin) ester). The absorbance was measured at 580 nm and 37 °C using a standard white lipase and a human physiological serum lipase. The amount of lipase units per liter (U/L) was calculated with the following equation:

(1) $$\text{Lipase activity}=\frac{\Delta A/{\text{min}}_{\text{sample}}}{\Delta A/{\text{min}}_{\text{calibrator}}}\times [\text{Calibrator}]$$

### 3.7. Determination of Range and Optimum Temperature

To determine the maximum efficiency temperature for the lipase activity of supernatant samples, a spectrophotometric system in a thermoregulation range of temperatures between 10 and 60 °C was used, and lipase activity was measured using a CPS-Controller thermal controller coupled with the SHIMADZU UV-123 spectrophotometer (Shimadzu Scientific Instruments, Columbia, MD, USA).

### 3.8. Determination of Detergent Tolerance

For tolerance of lipase activity in the presence of detergents, enzyme activity measurements were taken using the cationic detergent SDS and non-ionic detergents Tween-20 and Triton X-100 at three levels of concentration (1, 2, and 5 mM). All measurements were taken at 37 °C using the thermal controller CPS-Controller coupled with the SHIMADZU UV-123 spectrophotometer (Shimadzu Scientific Instruments, Columbia, MD, USA).

## 4. Conclusions

This study is the first report on a polypeptide from *Deschampsia antarctica* cloned in the *Pichia pastoris* system with lipase activity. The sequence cloned in the pPICZαB vector contained 97 amino acids that corresponded to 291 base pairs, as reported in GeneBank (Accession No. JX846628).

The recombinant lipase was expressed in X-33 cells, and a maximum activity of 35 U/L was registered at 37 °C and measured in the synthetic substrate 1,2-*o*-dilauryl rac-glycero-3-glutaric acid-(6-methylresorufin) ester. The detergent with minor inhibition of lipase activity was the cationic detergent, SDS, and in concentrations of between 1–5 mM, the relative activity was 52% with only small variations. Conversely, the relative activity of lipase enzymes in the non-ionic detergents TritonX100 and Tween 20 was decreased with high concentrations, reaching activity values of 22% and 16% at the end of experiment, respectively.
